# Microscopic Investigationsof Silicification and Lignification Suggest Their Coexistence in Tracheary Phytoliths in Date Fruits (*Phoenix dactylifera* L.)

**DOI:** 10.3389/fpls.2020.00977

**Published:** 2020-07-07

**Authors:** Navomy George, Asha Antony, Tholkappiyan Ramachandran, Fathalla Hamed, Afaf Kamal-Eldin

**Affiliations:** ^1^ Department of Food, Nutrition & Health, College of Food & Agriculture, United Arab Emirates University, Al-Ain, United Arab Emirates; ^2^ Department of Veterinary Medicine, College of Food & Agriculture, United Arab Emirates University, Al-Ain, United Arab Emirates; ^3^ Department of Physics, College of Science, United Arab Emirates University, Al-Ain, United Arab Emirates

**Keywords:** date fruits, silica, lignin, xylem vessels, sclereids, phytoliths

## Abstract

Date fruits are special representative of hard fruits and one of the richest sources of dietary silica and edible lignin, which are believed to have several health benefits. In this study, we used optical and scanning electron microscopy (SEM) to investigate the presence of associations between silicification and lignification in date fruits (*Phoenix dactylifera*, L.). Phloroglucinol staining was employed to observe lignification in date fruits, while silicification was studied by SEM of whole fruits and their acid digesta. This work revealed the presence of heterogeneity and complexity in the silica phytoliths and the lignified structures in date fruits. It was found that lignin exists independently of silica in the secondary cell walls of parenchymal and sclereid cells and that silica exists independently of lignin in the spheroid phytoliths that surround the sclereid cells. Interestingly, a small proportion of lignin and silica seemed to co-exist as partners in the spiral coils of the tracheid phytoliths.

## Introduction****

Date fruits (*Phoenix dactylifera*, Family: Palmae) are relatively dry fruits (moisture content, 10%–30%) with a high content of soluble sugars (60%–75%), accompanied by dietary fiber (5%–15%) and phenolic compounds (up to 5%) (Ghnimi et al., 2017). The dietary fiber composition of dates consists mostly of lignin, together with cellulose, hemicelluloses, and pectins (George et al., submitted). Lignin is a class of high-molecular-weight phenolic polymers that provide rigidity to xylem vessels (Michael, 2013). Date fruits are also rich sources of minerals, especially calcium, phosphorus, potassium, magnesium, iron, zinc, and cobalt (Habib and Ibrahim, 2011). In addition, a study reported that date fruits contained the highest concentration (0.02%) of dietary silica among 207 foods examined (Powell et al., 2005). Dietary soluble silicon is believed to provide several health benefits to consumers, including, *inter alia*, bone homeostasis and regeneration, stimulation of collagen synthesis, and skin, nail, and hair health (Martin, 2007; Zargar et al., 2012).

The study of silicification in the different organs and tissues of plants is an emerging field of research, e.g., regarding how and why plants assemble and use silicon. Biominerals such as silica are believed to provide plants with structural rigidity, mechanical strength, flexibility, as well as functional properties, including protection against biotic and abiotic stresses (Bauer et al., 2011). Silicon is absorbed by plants as water-soluble silicic acid (Si(OH)_4_), which, at a concentration that exceeds its solubility (>2 mM), starts to deposit as colloidal amorphous hydrated silica (SiO_2_.xH_2_O) and/or dehydrated form(s) of condensed polysilicic acid (SiO_x_(OH)_4–2x_) (Exley, 2015). Phytoliths are mineralized inorganic particles of silicon dioxide (SiO_2_) that are precipitated from the soluble monosilicic acid that is absorbed by plants from silica-rich soils (Eksambekar, 2009). Silica supplementation in grasses leads to the impregnation of cell walls with silica particles, which improves the mechanical properties of their tissues and increases their tolerance to biotic and abiotic stresses (Soukup et al., 2017).

Phytoliths are microscopic opal silica particles produced in many plants for variable functions (Madella et al., 2005). Date palm tissues (roots, stems, and leaves) contain up to 1% silicon, which is found as aggregates or phytoliths that are mainly associated with the sclerenchyma of the vascular bundles; moreover, the possible association between lignification and silica deposition was investigated but was ruled out (Bokor et al., 2019). In this study, we provide evidence that silicification and lignification are associated in the xylem vessels but not in the sclereid cells of the skin or parenchymal cell walls of date fruits.

## Materials and Methods

### Date Fruits

Emirati date fruits at the mature, Tamr stage, were received from the Al Foah date company (Alsaad, Abu Dhabi, UAE). After being received, the fruits were stored frozen and were thawed for at least 3 h before the experiments.

### Chemicals and Reagents

All chemicals and reagents used in this study were purchased from Sigma Aldrich (St. Louis, USA).

### Light Microscopy

The frozen dates were thawed, and samples of approximately 3 mm in length and breadth were cut from the fruits. The tissues were dipped in a graded series of ethanol (40%, 60%, and 80%) for 30 min each and finally kept in 80% ethanol overnight to enable the removal of sugars from the tissues and for fixation. The following morning, the pieces were washed again in 80% ethanol, followed by two washes in absolute ethanol and two washes (30 min and 1 h) in xylene. The pieces were embedded in paraffin, and radial sections of the fruits were obtained using a rotary microtome. The sections were placed in glass slides and were double-stained with safranin and Fast Green (Yilun et al., 1993), as follows. Aqueous safranin (1%) was added to the sections on the slide for about 2 min. Excess dye was then washed off with tap water, and the slide was rinsed in deionized water. Counterstaining was performed using 0.5% Fast Green in 95% ethanol for 5 min, after which the slides were rinsed thoroughly in tap water and deionized water to remove the excess stain. After wiping away the excess water, the slides were dried at 37°C in an oven for 30 min, the paraffin was removed from the sections in two changes of xylene (5 min each), and the dried sections were mounted with DPX. DPX—dibutylphthalate polystyrene xylene, is a mixture of distyrene (a plasticizer) and xylene that is used as a synthetic resin-based mounting medium in microscopic studies (Sigma Aldrich, St. Louis, USA). For lignin observation, the sections were stained with 1% phloroglucinol in 92% ethanol for 3 min and then transferred to 25% hydrochloric acid. Once total reddening of the specimens was achieved, the sections were immediately mounted with DPX.

### Scanning Electron Microscopy (SEM)

The dates were hand-cut into pieces of approximately 3 mm in length and breadth. The sugars were removed from the pieces by soaking in 80% ethanol (five times, 10 min each), and then dehydrated by two washes in acetone. The dehydrated pieces were mounted on aluminum studs using silver paint as an adhesive conductor. The pieces were sputter-coated with gold for observations. The SEM images of the fruit sections were obtained using an analytical scanning electron microscope (Jeol Analytical Scanning Electron Microscope, JEOL JSM-6010PLUS/LA, Tokyo, Japan). The scanning was performed at a low vacuum using a power of 20 kV, and the images were collected in the secondary electron imaging mode.

For microscopy of silica phytoliths, the date fruit pieces were digested in a mixture of equal volumes of concentrated sulfuric acid and nitric acid for at least 2 weeks. The resultant acid digesta were diluted with deionized water, and the precipitate was washed five times. The precipitate was then suspended in 95% ethanol and stored in vials. Observation of silica was carried out by placing a drop of the sample on the aluminum stud of the SEM stage. The ethanol solution was allowed to evaporate, and the stud was mounted onto the stage of the SEM for image acquisition. SEM/energy-dispersive X-ray spectroscopy (EDS) was employed to obtain the elemental mapping of the date fruit phytoliths.

## Results

### The Microstructure of Date Fruits

**Figure 1:**
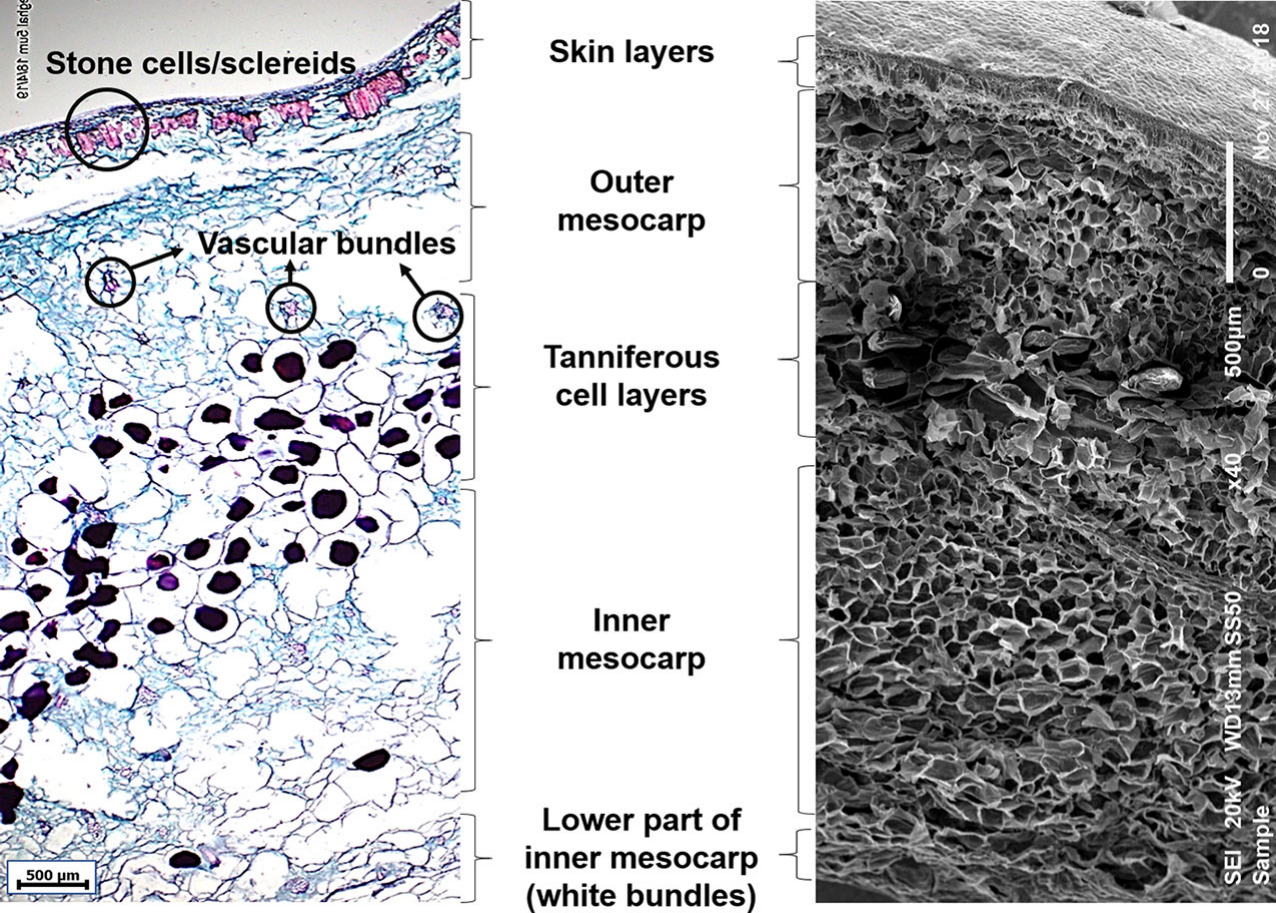
Microscopic images of mature date fruit showing cell layers from skin to inner mesocarp. Left: Light microscopic image showing various cell layers in a mature date fruit (5 µm thick section) stained with safranin and Fast Green. The lignified cells (sclereids and xylem vessels) are stained red, non-lignified phloem elements and parenchyma are stained bluish-green, and tannins are stained purple. Right: Scanning electron microscopic image of a mature date fruit showing various cell layers.

The microstructure of the edible part of the date fruit, as observed using optical microscopy, where various tissues take up different colors upon double staining with safranin and Fast Green is shown in **Figure 1**. Specifically, the lignin deposited in the sclereid cells and xylem vessels is stained with a pinkish-red color, while the tannins deposited in the vacuoles of the tanniferous layer are stained purple. Removal of the sugars from the fruit parenchymal cells leaves behind mainly the cell walls made of dietary fiber components (cellulose, hemicelluloses, and lignin), which are stained with a bluish-green color.

The date fruit exocarp (or skin) consists of one layer of epidermal cells covered by the cuticle and followed by two-to-three hypodermal cell layers and skin parenchymal layers. This is followed by an arrangement of distinct thick-walled sclerenchyma cells with a narrow cell lumen, called the stone cells or sclereids. The mesocarp, representing most of the fruit pulp and divided into outer and inner parts, consists of parenchymal cells and different levels of tanniferous middle layers (**Figure 1**) in agreement with Shomer et al. (1998). The outer and inner mesocarp regions are separated by three-to-seven layers of tanniferous cells, mainly consisting of condensed polyphenols or tannins as reported before (Shomer et al., 1998; Reuveni, 2018). Vascular bundles consisting of xylem and phloem elements are scattered in the outer and inner mesocarp in various sizes. The chemical removal of water and sugars from the fruit might have caused certain losses of cellular structure due to cell wall rupture, especially in the soft varieties (Coggins et al., 1967). The lower part of the inner mesocarp is the inner white edible portion of the date fruit, which consists of fibrous cells that are devoid of sugar (**Figure 1**).

### Lignification of Date Fruits

**Figure 2:**
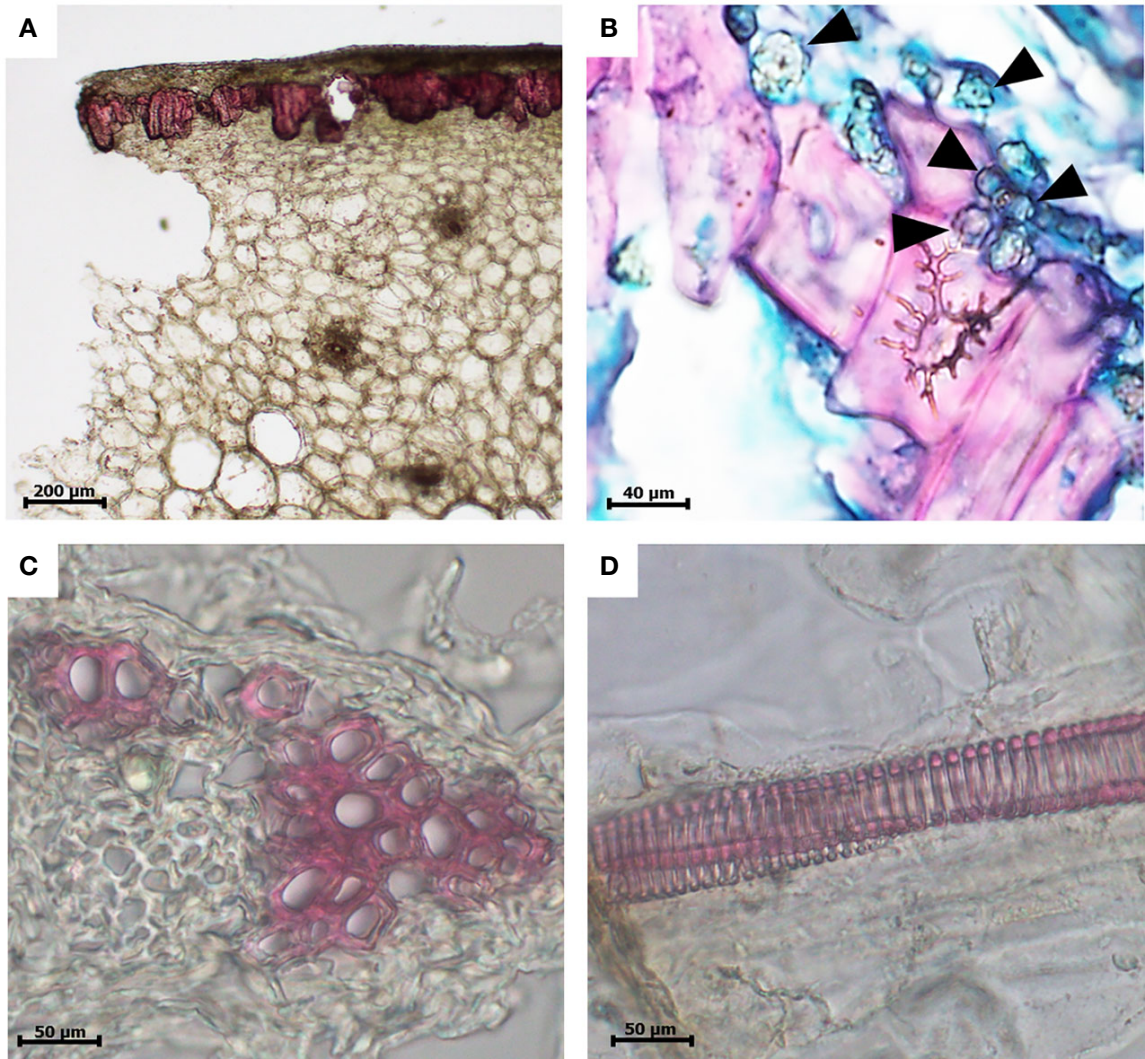
Light microscopic images of a 10-µm thick section of a date fruit stained with the lignin-specific phloroglucinol dye and showing lignin in bright red color. **(A)** Lignified sclereid cells (10×), **(B)** sclereid cells with secondary thickening of lignified cell walls surrounded by globular echinate phytoliths (100×) marked in the figure with black arrow heads, **(C)** xylem elements of the vascular bundles (40×), and **(D)** lignified xylem vessels (40×). 3(b) is double stained with safranin and Fast Green and not by phloroglucinol.

Light microscopy sections of date fruits stained with lignin-specific phloroglucinol, which stains lignin with a bright-red color, demonstrated that lignin was deposited in various cells, including the sclereids, the xylem elements of the vascular bundles, and the long xylem vessels of the mesocarp of the date fruit (**Figure 2A**). The sclereid cells in the skin parenchymal layer are shown in **Figure 2B**.

**Figure 3:**
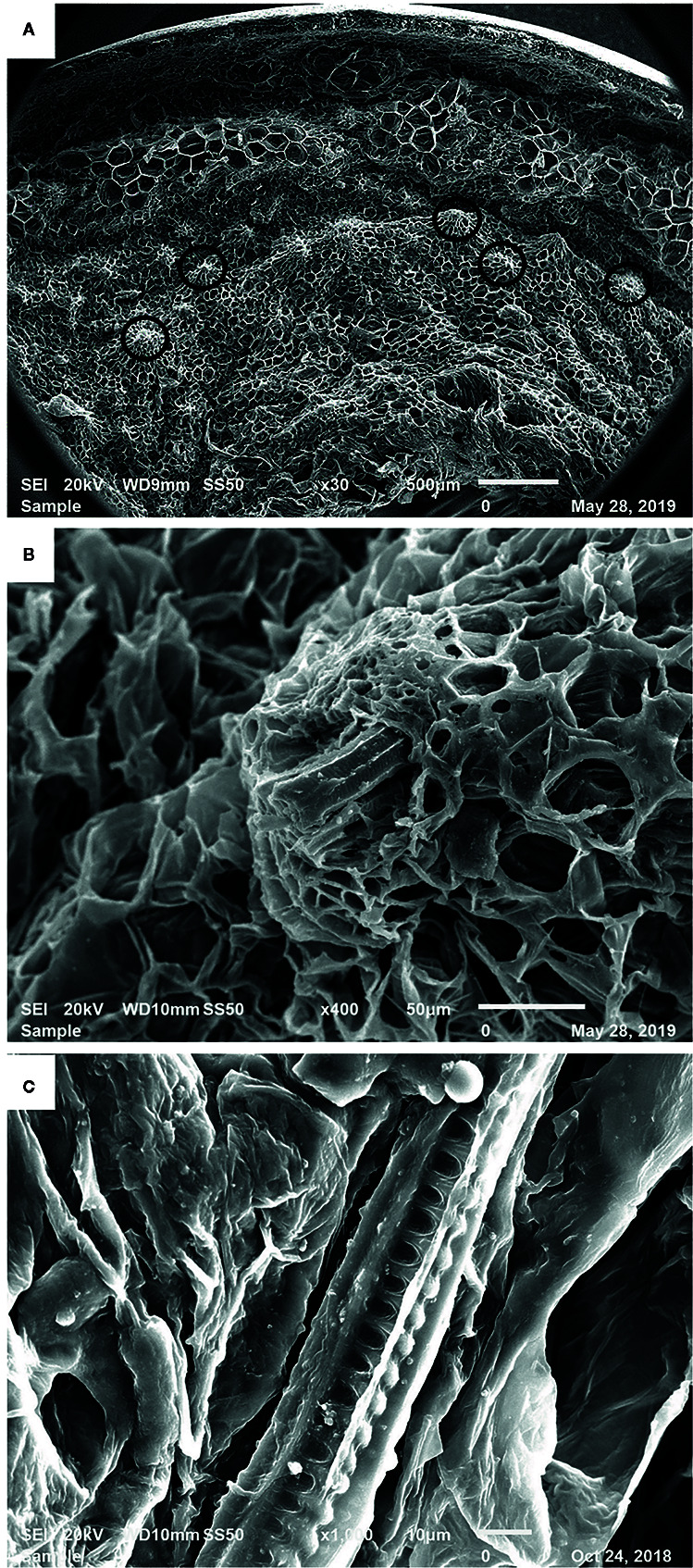
Scanning electron microscopic image of a mature date fruit showing **(A)** cell layers from skin to inner mesocarp (vascular bundles marked in black circles) **(B)** xylem of the vascular bundle among the parenchymal cells and **(C)** helical/spiral coils inside the xylem vessel.

**Figure 3** shows the SEM images of a mature date fruit section after the removal of sugars by ethanol washing, followed by dehydration with acetone. **Figure 3A** presents the various cell layers in a date fruit from skin to inner mesocarp with many vascular bundles observed in the mesocarp region (marked in black circles). A vascular bundle, with an intact xylem element surrounded by the phloem within the sugar-free parenchymal cells is shown in **Figure 3B**. Helical structures are clearly visible in **Figure 3C**.

### Silicification of Date Fruits

**Figure 4:**
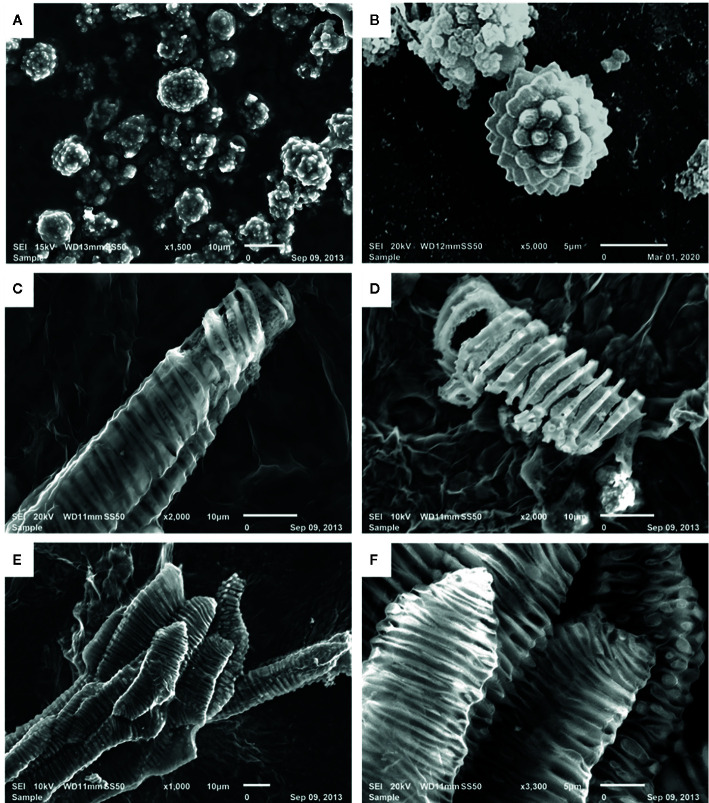
Scanning electron microscopic images of silica phytoliths: **(A, B)** spheroid echinate phytoliths, **(C, D)** tracheary annulate/helical phytoliths as helical coils with varying patterns on the helices, **(D)** vivid porous surface on a silica helix, and **(E, F)** articulated tracheary pitted phytoliths showing oval to circular perforations on the surface.

The digestion of date fruits in the concentrated acid mixture enables the removal of all digestible cell components leaving only silica and other ashes behind. **Figures 2B** and **4** depict the different manifestations of phytoliths, which are silicified plant bodies that were previously termed plant opals (ICPT, 2019) in date fruits. Two types of phytoliths were observed, i.e., spheroid and tracheary phytoliths. The observed spheroid silica phytoliths were exhibiting different sizes (**Figure 4A**). Moreover, they were echinate, with vivid, closely spaced petal-like projections that were arranged in a radiating fashion (**Figure 4B**). These phytoliths were abundant around the sclereid cells within the skin layer (**Figure 2B**). The second type of phytoliths observed in date fruits were tracheary annulate/helical phytoliths, which exhibited different shapes (**Figures 4C–F**) and were part of xylem vessels in the mesocarp. They consisted of a silicified outer wall and a hollow lumen and were relatively straight, cylindrical, and elongated, with a consistent diameter. Sometimes, branched structures were observed that exhibited straight, rounded, or pointed ends. Tracheary phytoliths with different types of surface ornamentations or surface textures occurred as single structures or in articulated groups. They exhibited microporous structures, sometimes with minute open pores on their surface.

**Figure 5:**
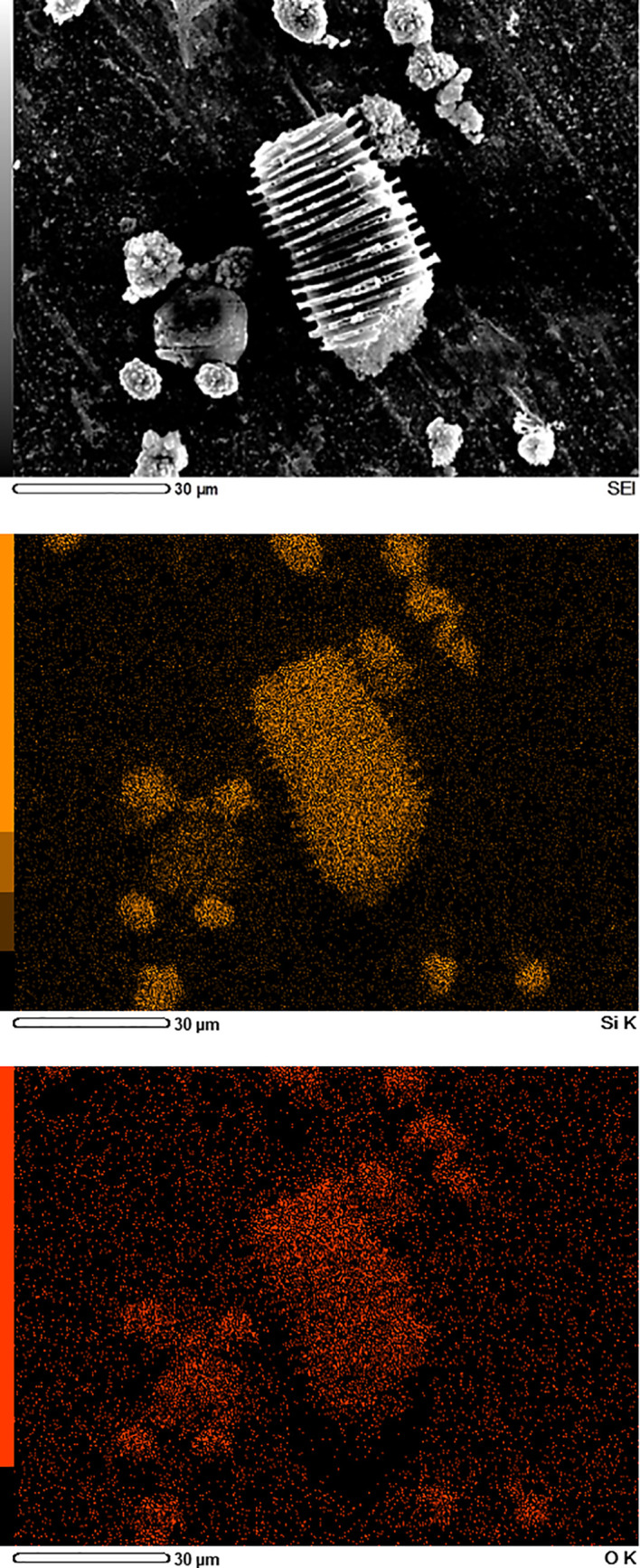
Scanning electron microscopy/energy-dispersive X-Ray spectroscopy (SEM/EDS) images of silica phytoliths in date fruit. The spheroid echinate and tracheary phytoliths obtained after digestion of a fruit sample with acid mixture contain only silicon (orange) and oxygen (red) in an atomic ratio of ca. 1:2, as shown by EDS mapping.

The elemental maps of the different phytoliths in acid digests of date fruits, i.e., spherical echinate and tracheary phytoliths, are depicted in **Figure 5**. These elemental maps show that the phytoliths are composed of silicon and oxygen in an atomic ratio of ca. 1:2, proving that the silica in phytoliths is deposited as SiO_2_.

## Discussion

In this study, we used optical and electron scanning microscopic techniques to assess the heterogeneity and complexity of the silicified and lignified structures within the microstructure of date fruits. The cell walls of parenchymal cells, the sclereids toward the skin of the fruit, and the walls of xylem vessels were highly lignified (**Figures 1** and **2**). Lignification, which is a highly controlled mechanism that starts at the early stages of tissue development, seems to play an important role in cell-wall thickening, thus providing rigidity, structural reinforcement, and flexibility to date fruits (Voxeur et al., 2015). Lignins are mainly made of *p*-coumaryl, coniferyl, and sinapyl alcohols, which give rise to *p*-hydroxyphenyl (H), guaiacyl (G), and sinapyl alcohol (S) monomeric units, respectively (Voxeur et al., 2015). Lignin is hydrophobic in nature, and it keeps the xylem vessels impermeable to water and assists in efficient transportation of water (Michael, 2013).

Although this has not been investigated further, date fruits were suggested to be the richest source of dietary silica (Jugdaohsingh et al., 2002; Powell et al., 2005). In this study, we observed two types of phytoliths in date fruits: spheroid phytoliths, which were concentrated mainly around the sclereid cells of the skin, and the tracheid phytoliths, located in the walls of the xylem vessels (**Figures 2** and **4**). The spheroid phytoliths of date fruits, which exhibited a typical surface morphology ranging from warty to echinate/speculate, are typical of date palm species (Prychid et al., 2003; Tomlinson et al., 2011; Bokor et al., 2019). The spheroid echinate phytoliths, which were previously termed globular echinates (ICPT, 2019), were abundant around the sclereid cells within the skin layers. The discovery of spheroid phytoliths in the teeth of Neanderthals in Iraq provided archeological evidence that date fruits were part of their diet (Hart, 2015). Here, we present for the first time the existence of tracheid phytoliths in date fruits. Rajat et al. (2019) reviewed articles published for around 40 years until 2017, and reported the presence of tracheid phytolith-type in a few plants of the Asteraceae family, e.g. in the leaves of *Helianthus annuus*, and the stem and leaves of *Brachylaena*, *Vernonia*, and *Aspilia* species. These types of phytoliths are commonly observed as multicelled silica skeletons with articulated structures in arid environments with high evapotranspiration rates (McCann, 1997; Rosen, 2008). The presence of silica in date fruits may contribute to the structure of their cells and fruits by increasing water-use efficiency and resistance to biotic and abiotic stresses (Bauer et al., 2011).

The similarity in spiral structure of lignin and silica phytoliths suggest their co-existence as components of the walls of the xylem vessels. The existence of a regulated association between silicification and lignification in plants has been suggested (Kang et al., 2019). Organic carbon was found to be trapped within some phytoliths, which may reflect an important role of the chemical environment in silica deposition (Perry and Keeling-Tucker, 2000; Parr and Sullivan, 2010; Gallagher et al., 2015). For example, studies of grasses suggested that lignification might be required for silica deposition (e.g. Zhang et al., 2013; Guerriero et al., 2016; Kumar et al., 2017; Soukup et al., 2017). The number of these phytoliths in date fruits is expected to be influenced by genetics (the cultivar) and agroclimatic conditions (soil, water supply, photosynthesis, *etc*.). The process of silicification is expected to be a hierarchical process that starts with the deposition of lignin on the hemicellulose–ferulic acid complex that is present in the secondary cell walls of plants. It was suggested that ferulic acid contributes to silicification *via* its attachment to the arabinoxylans that are present in grass cell walls and the anchoring of coniferyl alcohol, which initiates the deposition of lignin (Soukup et al., 2017). It was also suggested that mixed-linkage glucans play a controlling role by preventing the interactions between Si(OH)_4_ and arabinoxylan–ferulic acid complexes (Soukup et al., 2017). Conversely, the presence of silicon was suggested to have greater preference for organic polyhydroxyl compounds that are involved in lignin biosynthesis and to play a role in the production and accumulation of lignin in plants (Dorairaj and Ismail, 2017).

Here, we observed several associations between lignin and silica depositions in date fruits, namely the concentration of spheroid echinate phytoliths around the lignin-rich sclereid cells and the presence of possibly mixed spiral structures in the tracheary phytoliths in the xylem vessels. We showed that the spiral shapes of the silica structures of the tracheary phytoliths in the xylem vessels mimicked those of lignin, as visualized *via* specific staining within these vessels (**Figures 2D**, **3B**, and **4C–F**). Lignin deposition follows the polymerization of phenolic monolignol radicals, which is catalyzed by localized oxidative coupling enzymes, mainly class III peroxidases and laccases (Liberman and Benfey, 2013).

This study emphasized the presence of lignin in the secondary walls of parenchyma cells and sclereid cells, as well as the presence of silica in two different phytolith morphologies. The results of the study suggest that silica and lignin coexist as partners in the lignified xylem tracheids. Further understanding the supramolecular networks formed by the structural organization of carbohydrates, lignin, and silica in date fruits and their roles in the mechanical strength, rigidity, and protection against biotic and abiotic stresses will unravel important biochemical features of this fruit. As both lignin and silica may contribute to the hardness of date fruits, the regulation of their levels in this fruit deserves further studies to identify their specific roles and the mechanism(s) that drive their accumulation.

## Data Availability Statement

All datasets presented in this study are included in the article/supplementary material.

## Author Contributions

AK-E was in charge of the conceptualization of ideas, funding, and supervision of the study. NG, AA, and TR performed laboratory experiments, and FH evaluated the SEM results. NG and AK-E wrote the first draft of the manuscript. All authors contributed to the article and approved the submitted version.

## Funding

United Arab Emirates University (UPAR Grant 31F080).

## Conflict of Interest

The authors declare that the research was conducted in the absence of any commercial or financial relationships that could be construed as a potential conflict of interest.
